# Radiographic damage in early rheumatoid arthritis is associated with increased disability but not with pain—a 5-year follow-up study

**DOI:** 10.1186/s13075-023-03015-9

**Published:** 2023-02-27

**Authors:** Anna Eberhard, Emil Rydell, Kristina Forslind, Stefan Bergman, Thomas Mandl, Tor Olofsson, Lennart T. H. Jacobsson, Carl Turesson

**Affiliations:** 1 Rheumatology, Department of Clinical Sciences Malmö, Lund University, Jan Waldenströms gata 1b, 205 02 Malmö, Sweden; 2 Helsingborg Hospital, Helsingborg, Sweden; 3 Rheumatology, Department of Clinical Sciences Lund, Lund University, Lund, Sweden; 4 Spenshult Research and Development Center, Halmstad, Sweden; 5 Department of Public Health and Community Medicine, Institute of Medicine, Sahlgrenska Academy, University of Gothenburg, Gothenburg, Sweden; 6 Department of Rheumatology, Skåne University Hospital, Malmö, Sweden; 7 Department of Rheumatology and Inflammation Research, Sahlgrenska Academy, Gothenburg, Sweden

**Keywords:** Rheumatoid arthritis, Pain, Disability, Radiographic damage

## Abstract

**Objectives:**

To evaluate how radiographic damage, overall and measured as joint space narrowing score (JSNS) and erosion score (ES), as well as other clinical and laboratory measures, relate to disability and pain in early rheumatoid arthritis (RA).

**Methods:**

An inception cohort of 233 patients with early RA, recruited in 1995–2005, was followed for 5 years. Disability was assessed with the Health Assessment Questionnaire (HAQ), and pain with a visual analogue scale (VAS; 0–100 mm). Radiographs of hands and feet were evaluated using the Sharp-van der Heijde score (SHS), including JSNS and ES. The relation for radiographic scores and other clinical parameters with pain and HAQ were evaluated cross-sectionally by multivariate linear regression analysis and over time using generalized estimating equations.

**Results:**

ES was significantly associated with HAQ cross-sectionally at inclusion, after 2 and after 5 years, and over time. Associations for HAQ with SHS and JSNS were weaker and less consistent compared with those for ES. There was no association between radiographic scores and pain at any visit. Both HAQ and pain were associated with parameters of disease activity. The strongest cross-sectional associations were found for the number of tender joints (adjusted *p*<0.001 at all visits).

**Conclusion:**

Joint damage was associated with disability already in early RA. Erosions of hands and feet appear to have a greater influence on disability compared with joint space narrowing early in the disease. Pain was associated with other factors than joint destruction in early RA, in particular joint tenderness—suggesting an impact of pain sensitization.

**Supplementary Information:**

The online version contains supplementary material available at 10.1186/s13075-023-03015-9.

## Introduction

Rheumatoid arthritis (RA) is a disease characterized by inflammation of joints, which in time may result in joint destruction. Irreparable damage may occur to affected joints and can lead to increased disability. The Health Assessment Questionnaire (HAQ) is a validated measure for disability in RA [1]. Pain is another major symptom of RA and is typically associated with active inflammation. Although treatment outcome has greatly improved in RA in recent years [2], a substantial group of patients still suffer from non-resolved pain [3–6]. Both pain and HAQ have been associated with more sick leave, reduced quality of life, and worse mental health in patients with RA [7–9]. They are in other words factors that have a great impact on patients’ general health and well-being.

Joint destruction in RA includes damage to periarticular bone and articular cartilage, which can be identified by radiographs of hands and feet. Radiographic scoring can be used to assess the level of joint destruction and is often evaluated with composite measures like the Sharp-van der Heijde score (SHS) [10]. In the SHS, bone damage is quantified by the erosion score (ES) while the joint space narrowing score (JSNS) assesses damage to the cartilage.

RA-related disability is complex and could be explained both by current inflammation, which is known to fluctuate over time, and by joint destruction, which is more progressive and permanent. Several studies in established RA have shown that joint damage is associated with increased disability [11–13]. In patients with short disease duration, these associations have been less clear, whereas an association between radiographic scores and disability has repeatedly been shown in later stages of RA [14–16].

Several studies have assessed the association between disability and composite radiographic measures like the SHS, but only a few have investigated the effects of ES and JSNS separately. In these studies, disability displayed a stronger association with JSN than with erosions [17, 18].

Like disability, pain is a multifactorial experience, associated partly with disease activity, but also with central sensitization of the nervous system and psychologic factors [19]. It would also be reasonable to assume that joint destruction could have an impact on pain. This relationship is, however, less explored as compared with the association between joint damage and disability. Among patients with established RA in remission, MRI bone erosion, JSNS, and combined damage scores were associated with both HAQ and pain scores [20]. Another report concluded that MRI detected inflammation of the wrist, but not damage, was associated with functional impairment and pain in patients with early RA followed for 5 years [21].

Further insight into the relation for joint damage and other clinical measures with pain and disability in early disease could aid in early treatment decisions, with the goal of improving patient outcome. The purpose of this study was therefore to evaluate how joint damage, measured as SHS, as well as JSNS and ES separately, and other clinical and laboratory parameters, relates to pain and disability in early RA.

## Methods

### Patients

An inception cohort of 233 patients with early RA with a symptom duration ≤12 months was investigated. Patients were recruited in 1995–2005 from the rheumatology outpatient clinic of Malmö University Hospital, the only hospital serving the city, and from the four rheumatologists in private practice in Malmö. The patients were diagnosed by a specialist in rheumatology and fulfilled the 1987 American College of Rheumatology (ACR) classification criteria for RA [22]. Post hoc analyses indicated that the 2010 ACR/European League Against Rheumatism (EULAR) classification criteria for RA [23] were fulfilled by at least 88% of the patients [3]. The study was approved by the Regional Ethical Review Board for Southern Sweden, and all patients gave their written informed consent to participate in the study.

### Clinical assessment

Patients were followed according to a structured program with clinical examinations, laboratory tests, and radiographs of hands and feet at inclusion and 12, 24, and 60 months. All clinical examinations at all visits were performed by the same experienced rheumatologist for all patients. Patients were managed according to standard care with no pre-specified protocol for anti-rheumatic treatment. Assessment of pain (see Additional file 1) and the patients’ assessment of global disease activity (PGA) were evaluated with the visual analogue scale (VAS; 0–100 mm). Disability was assessed with the Swedish-validated version of the Stanford Health Assessment Questionnaire (HAQ) [24]. Information on height and weight was collected at inclusion and at the 2-year follow-up through a self-administered questionnaire. Disease activity was measured with the Disease Activity Score in 28 joints (DAS28). Information on current anti-rheumatic treatment was obtained at every visit through structured interviews. In addition, data on treatment with biologic DMARDs was obtained through linkage to a regional biologics register with 95% coverage in the area [25].

Erythrocyte sedimentation rate (ESR) and C-reactive protein (CRP) were measured with standard methods at the Department of Clinical Chemistry at Malmö University Hospital. IgM rheumatoid factor (RF) was analyzed using ELISA, which was calibrated against the World Health Organization RF reference preparation. Anti-cyclic citrullinated peptide (anti-CCP) antibodies were analyzed using the Quanta Lite CCP IgG ELISA (INOVA Diagnostics, USA).

Radiographs of hands and feet were scored according to the SHS [10], as previously described [26]. One trained reader, blinded to clinical information, scored all radiographs for SHS, including the sub-scores ES and JSNS. The maximum score for hands and feet is 448 for SHS, 280 for ES, and 168 for JSNS.

### Statistical analysis

Normality distribution of descriptive data was assessed with the Shapiro-Wilk test. Descriptive data was presented as mean (standard deviation) or median (interquartile range) depending on the distribution. Potential associations between each independent variable at inclusion and 1, 2, and 5 years and the outcome variables VAS pain and HAQ, at the same time points, were estimated with univariate linear regression analysis. Normality distribution of residuals was also evaluated with the Shapiro-Wilk test. All analyses were, in addition, performed with adjustment for sex and age. For the multivariate models, covariates were chosen based on the age- and sex-adjusted analysis, where covariates with a *p*-value ≤0.10 were considered for inclusion. In case of collinearity (bivariate correlation between covariates with *r* > 0.3), only the covariate with the strongest association with the outcome variable was included in the multivariate linear regression model. Sensitivity analyses were also performed with adjustment for the year of inclusion.

In addition, the longitudinal relation between radiographic and clinical parameters and outcomes was modeled using generalized estimating equations (GEE). With repeated measures of VAS pain or HAQ as the dependent variable, we investigated whether time-varying SHS, JSN, ES, and clinical variables were associated with worse pain or greater disability over time. These analyses were adjusted for age at inclusion and sex. Multivariate models were constructed based on principles similar to the linear regression analyses. All statistical analyses were performed using IBM SPSS statistics version 26.

## Results

### Patients

A total of 233 patients (median symptom duration 7 months) were investigated in this study (Table 1). VAS pain and HAQ improved from inclusion to 12 months. VAS pain was thereafter more or less unchanged during the follow-up period, while HAQ increased from 1 to 5 years (Table 1). Radiographic scores increased gradually between follow-up visits. Most patients were treated with methotrexate during the follow-up period, and 17% received a biologic DMARD at some point during the study.

**Table 1:** Patient characteristics at inclusion and at follow-up visits

Characteristic	Inclusion	1 year	2 years	5 years
*N*	232	219	208	179
Sex, female, *n* (%)	169 (70.3)	155 (70.8)	146 (70.2)	127 (70.9)
Age, years, mean (SD)	60.5 (14.6)	60.6 (14.6)	61.5 (14.9)	63.7 (14.6)
Symptom duration at inclusion, months	7.0 (5.0–10.0)	7.0 (5.0–10.0)	7.0 (5.0–10.0)	7.0 (5.0–10.0)
RF positive at inclusion, *n* (%)	143 (61.6)	135 (61.6)	125 (60.1)	115 (64.2)
Anti-CCP positive at inclusion, *n*/*N* (%)	116/202 (57.4)	109/189 (57.7)	102/180 (56.7)	91/155 (58.7)
Prednisolone, *n* (%)	90 (38.8)	69 (31.5)	63 (30.3)	52 (29.1)
Methotrexate, *n* (%)	124 (53.4)	137 (62.6)	128 (61.5)	110 (61.5)
Biologic DMARD, *n* (%)	0 (0)	12 (5.5)	17 (8.2)	32 (17.9)
>1 csDMARD, *n* (%)	4 (1.72)	14 (6.4)	20 (9.6)	16 (8.9)
No csDMARD, *n* (%)	41 (17.7)	28 (12.8)	36 (17.3)	42 (23.5)
Body mass index, mean (SD)	25.4 (4.2)^a^	NA	25.9 (4.5)^b^	NA
VAS pain, mean (SD)	41.2 (26.8)	30.1 (24.1)	32.1 (27.0)	30.3 (23.8)
DAS28, mean (SD)	4.6 (1.4)	3.7 (1.4)	3.6 (1.4)	3.6 (1.4)
SJC28	7.0 (5.0–11.0)	4.0 (2.0–7.0)	4.0 (2.0–7.0)	4.0 (2.0–7.0)
TJC28	4.0 (1.0–9.0)	2.0 (0–5.0)	1.0 (0–4.0)	1.0 (0–3.0)
HAQ	0.8 (0.4–1.3)	0 (0–1.0)	0.5 (0–1.0)	0.8 (0.1–1.1)
CRP (mg/l)	9.0 (<9–26.8)	<9 (<9–10.0)	<9 (<9–11.0)	<9 (<9–9.3)
CRP >9 mg/l, *n* (%)	121 (52.2)	58 (26.5)	62 (29.8)	44 (24.7)
ESR (mm/h)	20.5 (10.0–43.0)	15.0 (8.0–27.0)	15.0 (8.0–26.3)	15.0 (9.0–24.0)
VAS PGA, mean (SD)	43.3 (26.7)	30.6 (23.9)	33.6 (26.5)	34.5 (24.7)
SHS	3.0 (0–8.0)^c^	6.0 (1.0–16.0)^d^	10.0 (3.0–21.0)^e^	18.0 (6.0–31.0)^f^
ES	0 (0–2.0)^g^	1.0 (0–4.0)h	2.0 (0–6.0)^i^	5.0 (1.0–10.0)^j^
JSNS	2.0 (0–6.0)^k^	4.0 (0–12.0)^l^	6.0 (2.0–15.0)^m^	12.0 (3.0–23.0)^n^

Values are median (interquartile range) unless otherwise indicated. ^a^Data for body mass index in 162 cases. ^b^Data in 139 cases. ^c^Data for SHS in 217 cases. Range: 0–57. ^d^Data in 207 cases. Range: 0–86. ^e^Data in 195 cases. Range: 0–108. ^f^Data in 171 cases. Range: 0–165. ^g^Range: 0–22. ^h^Range: 0–41. ^i^Range: 0–53. ^j^Range: 0–94. ^k^Range: 0–45. ^l^Range: 0–68. ^m^Range: 0–77. ^n^Range: 0–74. *SD* standard deviation, *RF* rheumatoid factor, *Anti-CCP* anti-cyclic citrullinated peptide, *DMARD* disease-modifying anti-rheumatic drug, *csDMARD* conventional synthetic DMARD, *NA* not available, *VAS* visual analogue scale, *DAS28* Disease Activity Score in 28 joints, *SJC28* swollen joint count in 28 joints, *TJC28* tender joint count in 28 joints, *HAQ* Health Assessment Questionnaire, *CRP* C-reactive protein, *ESR* erythrocyte sedimentation rate, *PGA* patient global assessment, *SHS* Sharp-van der Heijde score, *ES* erosion score, *JSNS* joint space narrowing score

### Associations for clinical and radiographic parameters with VAS pain

#### Cross-sectional analysis

##### Univariate

In univariate linear regression analysis, there were no significant associations between radiographic scores and pain at any visit. However, positive significant associations between pain and SJC28, TJC28, ESR, and CRP were present at inclusion and at follow-up visits at 1, 2, and 5 years (Table 2). Furthermore, there was a significant association between pain and female sex at 2 years, and there was a negative association between pain and age (Table 2). The results were similar in the analysis adjusted for age and sex (Additional file 2, where details on covariate selection for multivariate analyses are given). There was no association between pain and body mass index (BMI) at inclusion or after 2 years (Table 2).

**Table 2:** Relation between clinical and radiographic parameters and VAS pain: univariate linear regression

Variable	Inclusion	1 year	2 years	5 years
	***β*** (95% CI)	***β*** (95% CI)	***β*** (95% CI)	***β*** (95% CI)
Female sex	−2.12 (−9.71, 5.46)	1.44 (−5.63, 8.50)	**11.89 (3.95, 19.81)**	6.16 (−1.55, 13.86)
Age (per year)	**−0.27 (−0.51, −0.04)**	−0.21 (−0.43, 0.01)	**−0.28 (−0.53, −0.03)**	0.04 (−0.21, 0.28)
Symptom duration (months)	**−1.67 (−2.84, −0.50)**	0.34 (−0.77 – 1.45)	0.18 (−1.10, 1.46)	0.24 (−0.99, 1.46)
Body mass index (per kg/m^2^)	0.04 (−0.97, 1.04)	-	0.43 (−0.61, 1.47)	-
RF seropositivity	−1.23 (−8.36, 5.90)	3.06 (−3.54, 9.66)	6.65 (−0.86, 14.16)	1.30 (−6.07, 8.63)
Anti-CCP seropositivity	−1.61 (−9.10, 5.89)	5.47 (−1.67, 12.60)	2.69 (−5.58, 10.96)	−1.64 (−9.52, 6.23)
SJC28	**1.34 (0.67, 2.01)**	**1.90 (1.15, 2.66)**	**1.71 (1.00, 2.42)**	**1.85 (1.18, 2.52)**
TJC28	**1.41 (0.91, 1.91)**	**2.25 (1.63, 2.88)**	**2.19 (1.55, 2.83)**	**2.02 (1.39, 2.65)**
ESR (per mm/h)	**0.27 (0.14, 0.40)**	**0.35 (0.19, 0.51)**	**0.47 (0.27, 0.67)**	**0.34 (0.08, 0.60)**
CRP (per mg/l)	**0.27 (0.17, 0.38)**	**0.34 (0.16, 0.51)**	**0.39 (0.15, 0.64)**	**0.48 (0.25, 0.70)**
SHS	−0.29 (−0.69, 0.10)	−0.02 (−0.26, 0.26)	0.12 (−0.11, 0.35)	0.05 (−0.09, 0.19)
ES	−0.12 (−1.39, 1.15)	0.22 (−0.48, 0.92)	0.44 (−0.11, 1.00)	0.22 (−0.08, 0.53)
JSNS	−0.44 (−0.94, 0.06)	−0.08 (−0.39, 0.23)	0.09 (−0.22, 0.40)	0.01 (−0.21, 0.23)

Bold text indicates statistical significance with *p*-values <0.05. *VAS* visual analogue scale, *CI* confidence interval, RF rheumatoid factor, *Anti-CCP* anti-cyclic citrullinated peptide, *SJC28* swollen joint count in 28 joints, *TJC28* tender joint count in 28 joints, *CRP* C-reactive protein, *ESR* erythrocyte sedimentation rate, *SHS* Sharp-van der Heijde score, *ES* erosion score, *JSNS* joint space narrowing score

##### Multivariate

In multivariate analysis, the associations with pain remained significant for TJC28 and CRP at inclusion and at 5 years and for TJC28 and ESR at 1 and 2 years. In addition, there were significant associations for female sex and lower age with pain in the multivariate analysis at 2 years (Table 3). The *r*-square for the multivariate models ranged from 0.24 to 0.29. Adjustment for the year of inclusion did not have a major impact on the results (Additional file 3).

**Table 3:** Relation for clinical and radiographic parameters with VAS pain and HAQ: multivariate linear regression

Dependent variable	Independent variable	*β*	95% CI	*p*-value	*r*-square
VAS pain					
		**Inclusion**			0.24
	Female	−2.68	−9.46–4.10	0.44	
	Age	−0.35	−0.56 to (−0.13)	<0.01	
	TJC28	1.18	0.70–1.66	<0.001	
	CRP	0.26	0.16–0.36	<0.001	
	Symptom duration	−0.68	−1.78–0.42	0.22	
		**1 year after inclusion**			0.24
	Female	−2.04	−8.37–4.29	0.53	
	Age	−0.16	−0.36–0.03	0.10	
	TJC28	1.97	1.34–2.60	<0.001	
	ESR	0.28	0.13–0.42	<0.001	
		**2 years after inclusion**			0.29
	Female	9.07	1.91–16.24	0.01	
	Age	−0.26	−0.49 to (−0.03)	0.03	
	TJC28	1.71	1.06–2.36	<0.001	
	ESR	0.40	0.20–0.59	<0.001	
	RF seropositivity	4.50	−2.62–11.62	0.21	
	ES	−0.03	−0.53–0.48	0.92	
		**5 years after inclusion**			0.24
	Female	3.41	−3.62–10.45	0.34	
	Age	−0.01	−0.22–0.21	0.95	
	TJC28	1.75	1.11–2.39	<0.001	
	CRP	0.37	0.15–0.58	0.001	
HAQ					
		**Inclusion**			0.39
	Female	0.177	0.028–0.326	0.02	
	Age	0.001	−0.004–0.006	0.63	
	TJC28	0.039	0.028–0.050	<0.001	
	CRP	0.007	0.005–0.009	<0.001	
	ES	0.032	0.006–0.057	0.02	
	Symptom duration	−0.020	−0.044–0.005	0.11	
		**2 years after inclusion**			0.30
	Female	0.222	0.049–0.395	0.01	
	Age	0.003	−0.002–0.009	0.22	
	TJC28	0.040	0.024–0.055	<0.001	
	ESR	0.010	0.006–0.015	<0.001	
	ES	0.010	−0.002–0.022	0.09	
		**5 years after inclusion**			0.28
	Female	0.292	0.091–0.492	<0.01	
	Age	0.012	0.005–0.018	<0.001	
	TJC28	0.037	0.018–0.055	<0.001	
	ESR	0.003	−0.004–0.010	0.43	
	ES	0.009	0.001–0.017	0.02	

*VAS* visual analogue scale, *CI* confidence interval, *RF* rheumatoid factor, *TJC28* tender joint count in 28 joints, *CRP* C-reactive protein, *ESR* erythrocyte sedimentation rate, *ES* erosion score, *HAQ* Health Assessment Questionnaire

#### Longitudinal analysis

In the longitudinal analysis using GEE, there were no significant associations for radiographic scores with pain over time (Table 4). Among variables significantly associated with pain over time in univariate analysis, age, ESR, and SJC28 remained significant also in multivariate analysis (Table 4).

**Table 4:** Relation between clinical and radiographic parameters and VAS pain: generalized estimating equations

Variable	Crude	Adj. for sex and age	Multivariate model
	***β*** (95% CI)	***β*** (95% CI)	***β*** (95% CI)
Female sex	2.96 (−2.01, 7.93)	−	3.63 (−1.11, 8.37)
Age (per year)	**−0.19 (−0.36, −0.01)**	−	**−0.26 (−0.43, −0.09)**
Symptom duration (months)	−0.46 (−1.28, 0.37)	−0.56 (−1.39, 0.28)	−
RF seropositivity	1.70 (−3.21, 6.61)	1.44 (−3.49, 6.37)	−
Anti-CCP seropositivity	−1.28 (−4.06, 6.62)	1.43 (−3.81, 6.67)	−
SJC28	***1.90 (1.53, 2.27)***	***1.92 (1.55, 2.30)***	***1.59 (1.21, 1.96)***
TJC28	***1.79 (1.49, 2.10)***	***1.78 (1.47, 2.08)***	−
ESR (per mm/h)	***0.41 (0.32, 0.51)***	***0.43 (0.34, 0.52)***	***0.34 (0.24, 0.43)***
CRP (per mg/l)	***0.36 (0.27, 0.44)***	***0.37 (0.29, 0.45)***	−
SHS	−0.04 (−0.18, 0.10)	−0.02 (−0.15, 0.12)	−
ES	−0.10 (−0.22, 0.43)	0.12 (−0.19, 0.43)	−
JSNS	−0.12 (−0.30, 0.05)	−0.09 (−0.27, 0.09)	−

Bold text indicates statistical significance with *p*-values <0.05. Italic text indicates *p*-values ≥0.05 and <0.10. Bold plus italic text indicates *p*-values <0.001. *VAS* visual analogue scale, *CI* confidence interval, *RF* rheumatoid factor, *Anti-CCP* anti-cyclic citrullinated peptide, *SJC28* swollen joint count in 28 joints, *TJC28* tender joint count in 28 joints, *CRP* C-reactive protein, *ESR* erythrocyte sedimentation rate, *SHS* Sharp-van der Heijde score, *ES* erosion score, *JSNS* joint space narrowing score

### Associations for clinical and radiographic parameters with disability (HAQ)

Due to the skewed distribution of the residuals in models including HAQ at 1 year, analyses for associations between HAQ and each individual variable were only performed at inclusion, 2 years, and 5 years, since the logarithmic transformation of HAQ at 1 year did not result in a normal distribution of the residuals.

#### Cross-sectional analysis

##### Univariate

In the univariate analysis, there were positive significant associations between HAQ and ES, SJC28, TJC28, CRP, and ESR at inclusion, 2 years, and 5 years (Table 5). Furthermore, significant associations for SHS, JSNS, female sex, and higher age with HAQ were present at 5 years, and for female sex with HAQ at 2 years (Table 5). Out of the radiographic scores, the strongest and most consistent associations with HAQ were seen for ES (*p*<0.001 at 5 years), rather than for JSNS or total SHS. The results were similar in the age- and sex-adjusted analysis (Additional file 4, where details on covariate selection for multivariate analyses are given). BMI was not associated with worse disability at inclusion or after 2 years (Table 5).

**Table 5:** Relation between clinical and radiographic parameters and HAQ: univariate linear regression

Variable	Inclusion	2 years	5 years
	***β*** (95% CI)	***β*** (95% CI)	***β*** (95% CI)
Female sex	0.164 (−0.013, 0.340)	**0.269 (0.079, 0.459)**	**0.274 (0.062, 0.485)**
Age (per year)	0.004 (−0.002, 0.010)	0.004 (−0.002, 0.010)	**0.012 (0.005, 0.018)**
Symptom duration (months)	**−0.053 (−0.080, −0.026)**	−0.007 (−0.038, 0.023)	−0.008 (−0.042, 0.026)
Body mass index (per kg/m^2^)	0.015 (−0.009, 0.038)	0.014 (−0.011, 0.038)	-
RF seropositivity	−0.149 (−0.315, 0.017)	−0.101 (−0.281, 0.080)	−0.170 (−0.373, 0.032)
Anti-CCP seropositivity	−0.093 (−0.271, 0.084)	−0.144 (−0.341, 0.052)	−0.123 (−0.343, 0.097)
SJC28	**0.042 (0.026, 0.057)**	**0.039 (0.022, 0.056)**	**0.044 (0.025, 0.063)**
TJC28	**0.043 (0.032, 0.055)**	**0.050 (0.035, 0.065)**	**0.052 (0.034, 0.070)**
ESR (per mm/h)	**0.009 (0.006, 0.012)**	**0.013 (0.009, 0.018)**	**0.011 (0.004, 0.019)**
CRP (per mg/l)	**0.009 (0.006, 0.011)**	**0.010 (0.004, 0.016)**	0.005 (−0.001, 0.012)
SHS	0.005 (−0.005, 0.015)	0.005 (0, 0.011)	**0.007 (0.003, 0.011)**
ES	**0.048 (0.018, 0.078)**	**0.021 (0.008, 0.035)**	**0.016 (0.008, 0.024)**
JSNS	0.001 (−0.012, 0.013)	0.003 (−0.005, 0.011)	**0.010 (0.004, 0.016)**

Bold text indicates statistical significance with *p*-values <0.05. *HAQ* Health Assessment Questionnaire, *CI* confidence interval, *RF* rheumatoid factor, *Anti-CCP* anti-cyclic citrullinated peptide, *SJC28* swollen joint count in 28 joints, *TJC28* tender joint count in 28 joints, *CRP* C-reactive protein, *ESR* erythrocyte sedimentation rate, *SHS* Sharp-van der Heijde score, *ES* erosion score, *JSNS* joint space narrowing score

##### Multivariate

In the multivariate linear regression model, ES remained significantly associated with HAQ at inclusion and 5 years. Furthermore, there were significant associations for both female sex and TJC28 with HAQ at inclusion, 2 years, and 5 years and for age at 5 years (Table 3). Significant associations with HAQ remained also for CRP at inclusion and for ESR at 2 years (Table 3). In these multivariate models, the *r*-square ranged from 0.28 to 0.39. Adjustment for the year of inclusion did not have a major impact on the results (Additional file 5).

#### Longitudinal analysis

In the longitudinal univariate model, significant associations were found for ES with HAQ over time, but not for JSN or SHS with HAQ over time (Table 6). Among variables significantly associated with HAQ over time in the univariate analysis, ES, female sex, and ESR remained significant also in the multivariate analysis (Table 6).

**Table 6:** Relation between clinical and radiographic parameters and HAQ: generalized estimating equations

Variable	Crude	Adj. for sex and age	Multivariate model
	***β*** (95% CI)	***β*** (95% CI)	***β*** (95% CI)
Female sex	**0.228 (0.086, 0.370)**	-	**0.229 (0.002, 0.456)**
Age (per year)	**0.006 (0.001, 0.011)**	-	0.003 (−0.003, 0.009)
Symptom duration (months)	**−0.027 (−0.052, −0.003)**	**−0.029 (−0.052, −0.005)**	*−0.027 (−0.056, 0.002)*
RF seropositivity	−0.139 (−0.287, 0.009)	−0.101 (−0.245, 0.044)	-
Anti-CCP seropositivity	−0.092 (−0.252, 0.067)	−0.082 (−0.236, 0.072)	-
SJC28	***0.039 (0.030, 0.047)***	***0.039 (0.031, 0.048)***	*0.011 (−0.001, 0.022)*
TJC28	***0.038 (0.030, 0.045)***	***0.038 (0.031, 0.045)***	
ESR (per mm/h)	***0.009 (0.007, 0.012)***	***0.009 (0.007, 0.012)***	**0.004 (0.001, 0.008)**
CRP (per mg/l)	***0.007 (0.005, 0.010)***	***0.008 (0.005, 0.010)***	***-***
SHS	−0.060 (−0.194, 0.075)	0.002 (−0.001, 0.005)	-
ES	**0.009 (0.002, 0.016)**	**0.009 (0.002, 0.015)**	**0.010 (0.001, 0.020)**
JSNS	0.002 (−0.003, 0.007)	0.001 (−0.004, 0.006)	-

Bold text indicates statistical significance with *p*-values <0.05. Italic text indicates *p*-values ≥0.05 and <0.10. Bold plus italic text indicates *p*-values <0.001. *HAQ* Health Assessment Questionnaire, *CI* confidence interval, *RF* rheumatoid factor, *Anti-CCP* anti-cyclic citrullinated peptide, *SJC28* swollen joint count in 28 joints, *TJC28* tender joint count in 28 joints, *CRP* C-reactive protein, *ESR* erythrocyte sedimentation rate, *SHS* Sharp-van der Heijde score, *ES* erosion score, *JSNS* joint space narrowing score

## Discussion

In this 5-year follow-up longitudinal cohort study of patients with early RA, we found that disability, measured as HAQ, was associated with ES, cross-sectionally, at inclusion and 5 years in multivariate analysis. Notably, the association for ES with HAQ was stronger and more consistent compared with that for SHS and JSNS with HAQ, respectively. Radiographic damage was not associated with pain at any point during the 5 years. By contrast, pain was associated with tender joint count and CRP (or ESR depending on the time of the outcome) in the multivariate analyses. In longitudinal analyses, using GEE, significant associations were found for ES with HAQ over time, but not with pain over time.

The stronger and more consistent association for ES with HAQ, as compared to JSNS with HAQ that was found in this study, contrasts with a report from Smolen et al. where JSN progression was the only damage score found to be associated with physical function in patients with early RA (<3-year disease duration) [17]. As their study investigated patients randomized to either adalimumab plus methotrexate or monotherapy in the PREMIER trial [17], the discrepancy from our study could be due to differences in radiographic progression and clinical outcomes between patients on early aggressive treatment in the context of a clinical trial, and an observational study of patients managed according to usual care. Another study on patients with new-onset RA found that erosive damage of the wrist, but not cartilage destruction, demonstrated by JSN, was associated with functional disability after 5 years of tightly controlled disease [27]. Furthermore, damage to the wrist was found to be the most important determinant of disability when analyzing joint groups separately. The same pattern was found by Gherghe et al., but in their study, they also showed that JSNS in the metacarpophalangeal joints was more strongly associated with impaired function [18]. This report was, however, based on patients with established RA. The conflicting results could possibly be explained by the fact that different types of lesions are more important in determining disability depending on the joints that are affected. Most likely, both cartilage destruction and erosions are important in determining physical function, and possibly JSN becomes more important with longer disease duration.

In contrast with several studies which have demonstrated joint damage to be related to disability only later during the disease course [14, 15], we found that associations between ES and HAQ existed already at inclusion in this cohort of patients with early RA. The current results are congruent with Odegard et al. who found that radiographic damage contributes to impaired physical function both at early and late disease stages [28]. This would point to the importance of early, aggressive anti-rheumatic treatment in order to stop radiographic damage and its associated disability. The conflicting findings in the various studies might be explained by differences in radiographic progression between cohorts, depending on, e.g., differences in treatment and the proportion of patients with markers of poor prognosis at inclusion. In this cohort, patients who were included during the first years of the study were not treated with biologic DMARDs, and the radiographic progression for these patients might be greater than for patients who were included later. However, in the present study, adjustment for the year of inclusion did not have a relevant impact on the results, and associations between disability and erosions were found already at inclusion, close to the time of diagnosis. Furthermore, several previous studies which did not find a relationship between radiographic damage and HAQ until later in the disease course were performed in smaller populations, compared with our cohort, resulting in lower power, and radiographic changes were not evaluated as ES and JSNS separately but only as composite scores (total SHS or the Larsen score) [15, 29, 30]. In line with those studies, statistically significant associations between SHS and HAQ were not found until the 5-year follow-up in this cohort.

The clinical relevance of the association between ES and HAQ should also be discussed. For example, a one-unit increase in ES at 5 years was associated with a 0.009 increase in HAQ, and ES in the highest quartile at 5 years ranged from 10 to 94. The minimum clinically important difference for HAQ has been reported to be 0.22 [31]. An increase of 0.009 in HAQ per unit increase in ES would hence correspond to an estimated difference in HAQ between 0.09 and 0.85 among those with extensive erosions (highest quartile) and is thus likely to be clinically significant in a subset of patients.

The lack of association between radiographic damage and pain indicates that other factors are responsible for pain in early RA. Based on the results from this study, factors associated with current joint inflammation seem to play a more important role in explaining pain. The tender joint count on the other hand, which had the strongest cross-sectional association with pain throughout the study, does not necessarily reflect only inflammation, but could also represent other pain mechanisms such as central sensitization, often present in chronic pain conditions like fibromyalgia. Coury et al. found that patients with RA and concurrent fibromyalgia were less likely to experience joint destruction [32]. These patients also had a significantly higher BMI compared with those without fibromyalgia. A negative association between high BMI and radiographic progression in RA has also been found in several studies [33–35]. In the present study, there was however no association between BMI and pain, although it could be noted that few patients in this cohort had severe overweight, with a mean BMI of 25 at inclusion. To conclude, pain in this cohort appears to be mainly related to inflammation, but could also be explained by central sensitization, supported by the strong association between tender joints and pain. The lack of association between joint damage and pain indicates that this aspect does not seem to contribute to the overall pain picture. It is possible that the general level of radiographic damage in early disease, such as in this cohort, is not enough to increase pain, and an effect of joint damage on pain in patients with more pronounced radiographic changes may appear later in the course of the disease. However, the joint damage among our patients was large enough to affect the level of disability already at inclusion.

Limitations of the study include the relatively small sample size which affects statistical power, especially for the analyses at 5 years where more patients have been lost to follow-up. Additionally, the majority of patients were included before the implementation of a treat to target strategy and before the use of biologic DMARDs became the standard of treatment in severe RA. The results of this study may therefore not be fully applicable to patients diagnosed after this period, although the association at inclusion should be less period-sensitive. Another limitation was that the scoring of the radiographs was performed by one single reader, increasing the risk for misclassification, although good reproducibility was demonstrated in a subset of the cohort [33]. Such misclassification is likely to be non-differential and is therefore unlikely to have resulted in any spurious results. Factors that we have not been able to evaluate, and which could have an impact on pain and disability, include mental health, coping techniques, and socioeconomic status [36–38]. Adding these variables to the model might have given a better explanatory power of the outcomes as the *r*-square ranged from 0.24 to 0.39 in the multivariate models. Furthermore, the study only evaluated radiographic damage of the hands and feet, and joint damage of larger joints might also have an impact on disability and pain.

Strengths of this study include the systematic longitudinal follow-up from a defined period of time and a defined catchment area. Consequently, selection bias should not be a major issue in this study, and the results could be generalized to patients seen in clinical practice.

## Conclusion

In this cohort of patients with early RA, statistically significant associations were found between bone damage and disability, measured by HAQ, already at inclusion. Disability was more strongly and consistently associated with erosions, and less so with cartilage destruction, demonstrated by JSN. This suggests that erosions have a greater influence on disability in early disease, although cartilage damage also appears to have an impact on function in the longer run. Joint damage was not associated with patient-reported pain at any point during the 5-year follow-up, suggesting that other factors play the main role in the multifactorial pain spectrum seen in RA—for example the potential development of a coexisting chronic pain syndrome. Both pain and disability were associated with increased laboratory markers of inflammation at all time points, but even more so with joint tenderness—compatible with a role for pain sensitization.

## Supplementary Information

**Additional file 1.** Question for pain.

**Additional file 2.** Relation for clinical and radiographic parameters with VAS pain; linear regression, adjusted for age and sex.

**Additional file 3.** Relation for clinical and radiographic parameters with VAS pain; multivariate linear regression. Sensitivity analysis, including inclusion calendar year as covariate.

**Additional file 4.** Relation for clinical and radiographic parameters with HAQ; linear regression, adjusted for age and sex.

**Additional file 5.** Relation for clinical and radiographic parameters with HAQ; multivariate linear regression. Sensitivity analysis, including inclusion calendar year as covariate.

## Data Availability

The data underlying this article will be shared on reasonable request to the corresponding author.

## Abbreviations

RA: Rheumatoid arthritis
HAQ: Health Assessment Questionnaire
SHS: Sharp-van der Heijde score
JSNS: Joint space narrowing score
ES: Erosion score
ACR: American College of Rheumatology
EULAR: European League Against Rheumatism
PGA: Patient global assessment
VAS: Visual analogue scale
DAS28: Disease Activity Score in 28 joints
DMARD: Disease-modifying anti-rheumatic drug
ESR: Erythrocyte sedimentation rate
CRP: C-reactive protein
Anti-CCP: Anti-cyclic citrullinated peptide
GEE: Generalized estimating equations
SJC28: Swollen joint cout in 28 joints
TJC28: Tender joint count in 28 joints
BMI: Body mass index
