# Supplementary material for: Radiographic damage in early rheumatoid arthritis is associated with increased disability but not with pain—a 5-year follow-up study
Source: Arthritis Res Ther. 2023 Feb 27;25:29. doi: 10.1186/s13075-023-03015-9 (PMC9969673; doi:10.1186/s13075-023-03015-9)
Supplement: Supplementary file 1 — Additional file 1. Question for pain. [file 13075_2023_3015_MOESM1_ESM.docx]

**Additional file 1.**

*Question for pain*

How much pain did you have from your joint disease during the last week? The patients were asked to rate this on a visual analogue scale (0-100 mm), reaching from “inte ont alls” (no pain at all) to “värsta tänkbara smärta” (worst possible pain).
