# Supplementary material for: Radiographic damage in early rheumatoid arthritis is associated with increased disability but not with pain—a 5-year follow-up study
Source: Arthritis Res Ther. 2023 Feb 27;25:29. doi: 10.1186/s13075-023-03015-9 (PMC9969673; doi:10.1186/s13075-023-03015-9)
Supplement: Supplementary file 2 — Additional file 2. Relation for clinical and radiographic parameters with VAS pain; linear regression, adjusted for age and sex. [file 13075_2023_3015_MOESM2_ESM.docx]

**Additional file 2.**

Relation for clinical and radiographic parameters with VAS pain; linear regression, adjusted for age and sex

|  | Inclusion | 1 year | 2 years | 5 years |
| --- | --- | --- | --- | --- |
| Variable | **β (95% CI)** | **β (95% CI)** | **β (95% CI)** | **β (95% CI)** |
| RF seropositivity | -2.28 (-9.40, 4.85) | 2.43 (-4.22, 9.08) | *6.79 (-0.61, 14.20)* | 2.27 (-5.18, 9.71) |
| Anti-CCP seropositivity | -1.68 (-9.16, 5.79) | 5.35 (-1.73, 12.43) | 3.52 (-4.51, 11.55) | -1.16 (-9.08, 6.77) |
| Symptom duration | **-1.74 (-2.91, -0.58)** | 0.27 (-0.84, 1.38) | -0.04 (-1.30, 1.21) | 0.20 (-1.03, 1.43) |
| Body mass index | -0.02 (-1.02, 1.00) | - | 0.42 (-0.59, 1.43) | - |
| SJC28 | ***1.30 (0.62, 1.97)*** | ***1.90 (1.15, 2.65)*** | ***1.77 (1.08, 2.46)*** | ***1.84 (1.17, 2.52)*** |
| TJC28 | ***1.38 (0.88, 1.88)*** | ***2.22 (1.59, 2.85)*** | ***2.07 (1.44, 2.71)*** | ***1.98 (1.34, 2.62)*** |
| ESR | ***0.31 (0.18, 0.44)*** | ***0.36 (0.20, 0.52)*** | ***0.50 (0.30, 0.69)*** | **0.35 (0.09, 0.61)** |
| CRP | ***0.31 (0.20, 0.41)*** | ***0.35 (0.18, 0.52)*** | **0.41 (0.17, 0.65)** | ***0.49 (0.26, 0.72)*** |
| SHS | -0.13 (-0.56, 0.30) | 0.10 (-0.16, 0.35) | 0.18 (-0.05, 0.41) | 0.04 (-0.96, 0.18) |
| ES | 0.35 (-0.96, 1.67) | 0.40 (-0.30, 1.10) | *0.50 (-0.04, 1.03)* | 0.28 (-0.08, 0.53) |
| JSNS | -0.26 (-0.79, 0.28) | 0.06 (-0.27, 0.40) | 0.18 (-0.14, 0.50) | -0.01 (-0.23, 0.22) |

Bold text indicates statistical significance with p-values <0.05. Italic text indicates p-values <0.10. Bold plus italic text indicates p-values <0.001.
VAS: visual analogue scale, CI: confidence interval, RF: rheumatoid factor, Anti-CCP: anti-cyclic citrullinated peptide, SJC28: swollen joint count in 28 joints, TJC28: tender joint count in 28 joints, CRP: C-reactive protein, ESR: erythrocyte sedimentation rate, SHS: Sharp-van der Heijde score, ES: erosion score, JSNS: joint space narrowing score.
**Inclusion**: SJC28, TJC28, ESR, CRP had p <0.10, and were considered for multivariate analysis. ESR and SJC28 were excluded due to collinearity with CRP and TJC28, respectively.
**1 year**: SJC28, TJC28, ESR, CRP had p <0.10, and were considered for multivariate analysis. CRP and SJC28 were excluded due to collinearity with ESR and TJC28, respectively.
**2 years**: SJC28, TJC28, ESR, CRP, RF and ES had p <0.10, and were considered for multivariate analysis. CRP and SJC28 were excluded due to collinearity with ESR and TJC28, respectively.

**5 years**: SJC28, TJC28, ESR, CRP had p <0.10, and were considered for multivariate analysis. ESR and SJC28 were excluded due to collinearity with CRP and TJC28, respectively.
