# Supplementary material for: Radiographic damage in early rheumatoid arthritis is associated with increased disability but not with pain—a 5-year follow-up study
Source: Arthritis Res Ther. 2023 Feb 27;25:29. doi: 10.1186/s13075-023-03015-9 (PMC9969673; doi:10.1186/s13075-023-03015-9)
Supplement: Supplementary file 3 — Additional file 3. Relation for clinical and radiographic parameters with VAS pain; multivariate linear regression. Sensitivity analysis, including inclusion calendar year as covariate. [file 13075_2023_3015_MOESM3_ESM.docx]

**Additional file 3.**

Relation for clinical and radiographic parameters with VAS pain; multivariate linear regression. Sensitivity analysis, including inclusion calendar year as covariate.

VAS: visual analogue scale, CI: confidence interval, RF: rheumatoid factor, TJC28: tender joint count in 28 joints, CRP: C-reactive protein, ESR: erythrocyte sedimentation rate, ES: erosion score.

| Variable | β | 95% CI | P-value | R-square |
| --- | --- | --- | --- | --- |
|  | **Inclusion** |  |  | 0.24 |
| Female | -2.85 | -9.64 – 3.94 | 0.41 |  |
| Age | -0.33 | -0.55 – (-0.11) | <0.01 |  |
| Inclusion year | 0.53 | -0.74 – 1.79 | 0.74 |  |
| TJC28 | 1.17 | 0.69 – 1.65 | <0.001 |  |
| CRP | 0.25 | 0.14 – 0.36 | <0.001 |  |
| Symptom duration | -0.58 | -1.71 – 0.56 | 0.32 |  |
|  | **1 year after inclusion** | | | 0.24 |
| Female | -2.04 | -8.38 – 4.31 | 0.53 |  |
| Age | -0.16 | -0.36 – 0.04 | 0.11 |  |
| Inclusion year | 0.00 | -1.11 – 1.12 | 1.00 |  |
| TJC28 | 1.97 | 1.33 – 2.61 | <0.001 |  |
| ESR | 0.28 | 0.13 – 0.43 | <0.001 |  |
|  | **2 years after inclusion** |  |  | 0.29 |
| Female | 9.09 | 1.91 – 16.27 | 0.01 |  |
| Age | -0.26 | -0.49 – (-0.03) | 0.03 |  |
| Inclusion year | -0.14 | -1.39 – 1.11 | 0.83 |  |
| TJC28 | 1.70 | 1.05 – 2.36 | <0.001 |  |
| ESR | 0.40 | 0.20 – 0.59 | <0.001 |  |
| RF seropositivity | 4.54 | -2.61 – 11.69 | 0.21 |  |
| ES | -0.03 | -0.53 – 0.49 | 0.93 |  |
|  | **5 years after inclusion** |  |  | 0.24 |
| Female | 3.38 | -3.67 – 10.42 | 0.41 |  |
| Age | 0.00 | -0.22 – 0.22 | 0.98 |  |
| Inclusion year | 0.52 | -0.71 – 1.75 | 0.40 |  |
| TJC28 | 1.81 | 1.15 – 2.46 | <0.001 |  |
| CRP | 0.37 | 0.16 – 0.59 | 0.001 |  |
