# Supplementary material for: Radiographic damage in early rheumatoid arthritis is associated with increased disability but not with pain—a 5-year follow-up study
Source: Arthritis Res Ther. 2023 Feb 27;25:29. doi: 10.1186/s13075-023-03015-9 (PMC9969673; doi:10.1186/s13075-023-03015-9)
Supplement: Supplementary file 4 — Additional file 4. Relation for clinical and radiographic parameters with HAQ; linear regression, adjusted for age and sex. [file 13075_2023_3015_MOESM4_ESM.docx]

**Additional file 4.**

Relation for clinical and radiographic parameters with HAQ; linear regression, adjusted for age and sex

|  | Inclusion | 2 years | 5 years |
| --- | --- | --- | --- |
| Variable | **β (95% CI)** | **β (95% CI)** | **β (95% CI)** |
| RF seropositivity | -0.124 (-0.290, 0.043) | -0.064 (-0.243, 0.116) | -0.092 (-0.288, 0.104) |
| Anti-CCP seropositivity | -0.085 (-0.260, 0.089) | -0.128 (-0.631, 0.621) | -0.097 (-0.308, 0.114) |
| Symptom duration | ***-0.054 (-0.081, -0.027)*** | -0.010 (-0.040, 0.021) | -0.005 (-0.037, 0.028) |
| Body mass index | 0.019 (-0.004, 0.042) | 0.015 (-0.009, 0.039) | - |
| SJC28 | ***0.047 (0.032, 0.062)*** | ***0.038 (0.021, 0.055)*** | ***0.038 (0.019, 0.056)*** |
| TJC28 | ***0.044 (0.033, 0.055)*** | ***0.048 (0.033, 0.063)*** | ***0.048 (0.031, 0.065)*** |
| ESR | ***0.009 (0.006, 0.012)*** | ***0.013 (0.009, 0.018)*** | **0.010 (0.003, 0.017)** |
| CRP | ***0.009 (0.006, 0.011)*** | **0.010 (0.004, 0.016)** | 0.004 (-0.002, 0.011) |
| SHS | 0.002 (-0.009, 0.012) | 0.004 (-0.001, 0.010) | **0.006 (0.003, 0.010)** |
| ES | **0.042 (0.011, 0.073)** | **0.021 (0.008, 0.034)** | ***0.016 (0.008, 0.024)*** |
| JSNS | -0.004 (-0.017, 0.008) | 0.001 (-0.007, 0.009) | **0.007 (0.001, 0.013)** |

Bold text indicates statistical significance with p-values <0.05. Italic text indicates p-values <0.10. Bold plus italic text indicates p-values <0.001.
HAQ: health assessment questionnaire, CI: confidence interval, RF: rheumatoid factor, Anti-CCP: anti-cyclic citrullinated peptide, SJC28: swollen joint count in 28 joints, TJC28: tender joint count in 28 joints, CRP: C-reactive protein, ESR: erythrocyte sedimentation rate, SHS: Sharp-van der Heijde score, ES: erosion score, JSNS: joint space narrowing score.

**Inclusion**: SJC28, TJC28, ESR, CRP and ES had p <0.10, and were considered for multivariate analysis. ESR was excluded due to collinearity with CRP. There was collinearity between SJC28 and TJC28. As they had similar association with HAQ, TJC28 was selected for consistency with other time points.
**2 years**: SJC28, TJC28, ESR, CRP and ES had p <0.10, and were considered for multivariate analysis. CRP and SJC28 were excluded due to collinearity with ESR and TJC28, respectively.
**5 years**: SJC28, TJC28, ESR, ES, JSNS and SHS had p <0.10, and were considered for multivariate analysis. CRP and SJC28 were excluded due to collinearity with ESR and TJC28, respectively. SHS and JSNS were excluded due to collinearity with ES.
