# Supplementary material for: Radiographic damage in early rheumatoid arthritis is associated with increased disability but not with pain—a 5-year follow-up study
Source: Arthritis Res Ther. 2023 Feb 27;25:29. doi: 10.1186/s13075-023-03015-9 (PMC9969673; doi:10.1186/s13075-023-03015-9)
Supplement: Supplementary file 5 — Additional file 5. Relation for clinical and radiographic parameters with HAQ; multivariate linear regression. Sensitivity analysis, including inclusion calendar year as covariate. [file 13075_2023_3015_MOESM5_ESM.docx]

**Additional file 5.**

Relation for clinical and radiographic parameters with HAQ; multivariate linear regression.
Sensitivity analysis, including inclusion calendar year as covariate.

| Variable | β | 95% CI | P-value | R-square |
| --- | --- | --- | --- | --- |
|  | **Inclusion** |  |  | 0.40 |
| Female | 0.176 | 0.027 – 0.326 | 0.02 |  |
| Age | 0.002 | -0.003 – 0.007 | 0.54 |  |
| Inclusion year | 0.009 | -0.019 – 0.037 | 0.53 |  |
| TJC28 | 0.039 | 0.028 – 0.050 | <0.001 |  |
| CRP | 0.007 | 0.004 – 0.009 | <0.001 |  |
| ES | 0.030 | 0.004 – 0.056 | 0.02 |  |
| Symptom duration | -0.018 | -0.043 – 0.008 | 0.17 |  |
|  | **2 years after inclusion** | | | 0.30 |
| Female | 0.222 | 0.048 – 0.395 | 0.01 |  |
| Age | 0.004 | -0.002 – 0.009 | 0.21 |  |
| Inclusion year | 0.006 | -0.025 – 0.036 | 0.72 |  |
| TJC28 | 0.040 | 0.025 – 0.056 | <0.001 |  |
| ESR | 0.010 | 0.006 – 0.015 | <0.001 |  |
| ES | 0.010 | -0.002 – 0.022 | 0.10 |  |
|  | **5 years after inclusion** |  |  | 0.28 |
| Female | 0.292 | 0.091 – 0.494 | <0.01 |  |
| Age | 0.012 | 0.005 – 0.018 | <0.001 |  |
| Inclusion year | -0.002 | -0.037 – 0.033 | 0.91 |  |
| TJC28 | 0.036 | 0.017 – 0.055 | <0.001 |  |
| ESR | 0.003 | -0.004 – 0.010 | 0.43 |  |
| ES | 0.009 | 0.001 – 0.017 | 0.02 |  |

HAQ: health assessment questionnaire, CI: confidence interval, TJC28: tender joint count in 28 joints, CRP: C-reactive protein, ES: erosion score, ESR: erythrocyte sedimentation rate.
